# A New Electrochemical Detection Technique for Organic Matter Content in Ecological Soils

**DOI:** 10.3389/fchem.2021.699368

**Published:** 2021-06-25

**Authors:** Jinping Liu, Tao Yang, Jiaqi Xu, Yankun Sun

**Affiliations:** ^1^ College of Resources and Environment, Northeast Agricultural University, Harbin, China; ^2^ Heilongjiang Vocational College of Agricultural Technology, Jiamusi, China

**Keywords:** soil organic matter, cyclic voltammetry, graphene, equivalent model set, joint model

## Abstract

The rapid detection of organic matter in soil is of great interest in agriculture, but the commonly used techniques require laboratory operation. Therefore, the development of a technique that allows rapid detection of soil organic matter in the field is of great interest. In this work, we propose an electrochemical-based approach for the detection of organic matter in soil particles. Since soil particles immobilized directly on the electrode surface can fall off during testing, we introduced graphene to coat the soil particles. The encapsulated soil particles can be stably immobilized on the electrode surface. We have investigated the electrochemical behavior of soil particles. The results show a correspondence between the electrochemical oxidation and reduction of soil particles and the organic matter content in them. We collected soil samples from three sites and constructed an electrochemical modeling, testing framework with stability based on multiple calibrations and random division of the prediction set. We used the equal interval partial least squares (EC-PLS) method for potential optimization to establish the equivalent model set. A joint model for the electrochemical analysis of organic matter in three locations of soil samples was developed for the commonality study.

## Introduction

Soil organic matter content is one of the most important indicators of soil nutrient supply capacity and fertility. Soil research scholars generally agree that there is a significant positive correlation between soil organic matter content and soil quality. Therefore, the information of soil organic matter content is one of the important value in the evaluation of ecological environment. At present, the determination of soil organic matter content is generally performed in the laboratory by external heating with potassium dichromate and combined spectroscopic/chromatographic analytical techniques such as nuclear magnetic resonance and thermal cracking-mass spectrometry (Yang et al., 2011). These determination methods have the advantage of higher measurement accuracy, however, they require high operator requirements and longer time for detection, while a large amount of reagents are consumed during the monitoring process.

In recent years, near-infrared spectroscopy has rapidly developed into a rapid, nondestructive, multi-component simultaneous analysis technique. However, due to the complex composition of soils, the investigations realized that two factors affect the spectral reflectance properties of soils: the moisture content of the soil and iron-containing oxides. There is a high correlation between the moisture in the soil and the reflectance of the spectrum, and an increase in soil moisture content leads to a decrease in spectral reflectance (Summers et al., 2011). In addition, the presence of iron oxides causes a decrease in spectral reflectance and there are multiple characteristic absorption summits in the soil spectrum caused by iron oxides (Liu et al., 2018; Babaeian et al., 2019). Furthermore, the ensemble frequency multiplication information of the chemical bonds of hydrogen-containing groups contained in the NIR spectra is very weak, and it is generally necessary to pre-process the spectral data and then build a quantitative calibration model of the spectra by chemometric methods. Due to the presence of objective reasons such as the state of the sample, errors in measurement, and instrument errors, the raw spectra obtained from the instrument often contain some interference information that is not related to the components to be measured. The presence of interfering information can have an impact on subsequent calibration model building such as reduced accuracy or inaccurate prediction. Many studies are currently centered on the development of spectral processing algorithms to reduce the effects of interferences (Cambou et al., 2016; Sharififar et al., 2019). Meanwhile, some scholars have borrowed analytical techniques from other fields for detecting organic matter content in soils. For example, thermogravimetric analysis and differential scanning calorimetry, which are widely used in materials science, have been used for the evaluation of soil organic matter content (Fernández et al., 2012; Siewert and Kučerík, 2015). Wiesheu et al. (Wiesheu et al., 2018) explored the use of isotopic internal standards combined with Raman spectroscopy for the detection of soil organic matter content. These efforts provide very valuable academic implications, but still rely on large instruments in the detection results and require tedious processing of soil samples. In this work, we aim to explore an original detection technique based on solid state electroanalytical chemistry (SSEAC) for the rapid analysis of soil organic matter content in the field.

The application of electrochemical techniques in the field of soil science consists of two main categories: 1) electrochemical remediation of contaminated soils (Muñoz-Morales et al., 2017; Domínguez-Garay et al., 2018), and 2) detection of specific soil contaminants (Zhao and Liu, 2018; Mondal and Subramaniam, 2019). In the second application area, investigations have made a number of breakthroughs using liquid-phase electrochemical analysis techniques that allow specific and highly sensitive detection of pesticide residues and heavy metals in soils. However, it is not reasonable to design techniques for the detection of soil organic matter content using this type of methodology because the soil sample pre-treatment techniques for this type of technique are similar to conventional chemical assays, which rely on laboratory instruments and do not offer superiority. In this work, we propose an alternative approach to evaluate the organic matter content of undigested/dissolved soil samples directly by using the SSEAC technique for rapid detection. Soil particles contain a variety of plant-derived organic matter molecules, some of which are capable of electrochemical oxidation and/or reduction reactions, such as indoles, polyphenols and quinones. In particular, lignin and cross-linked phenolic polymers undergo substitution, addition, coupling and bond cleavage reactions in electrochemical oxidation and reduction. The electrochemical signal of these substances provides an indication that can be used to react to the overall organic matter content of the soil.

## Experiments

In the study of this paper, soil areas from three locations were collected (A, B, C). Each location was collected 77 soil samples. All soil samples will be used to determine the organic matter content of the soil using conventional and routine chemical methods, but some pretreatment of the soil samples will be required before measurement. At the beginning of the experiment, all the soil samples collected were numbered and recorded separately. The numbered soil samples were placed sequentially on vellum and spread evenly and neatly in a cool, dry and ventilated environment for air-drying. Particular care should be taken not to expose the soil samples to direct sunlight and baking during this process, as this can damage the integrity of the soil sample composition and affect the accuracy of the organic matter measurements. After the air-drying of all soil samples, the visible impurities are removed from the soil samples and the lumpy soil samples are carefully ground so that all soil samples are in a homogeneous fine-grained state. These samples are then passed through a 0.25 mm pore sieve, after which the soil samples are preserved for the subsequent determination of soil organic matter.

All electrochemical measurements were carried out using a CHI 760e working station. Then, the polydopamine functionalized graphene has been used for soil particle encapsulation. Then 2 μL of the dispersion was drop coated on a glassy carbon electrode and used as working electrode. A Pt wire and an Ag/AgCl electrode have been used as counter electrode and reference electrode, respectively.

## Results and Discussion

**FIGURE 1:**
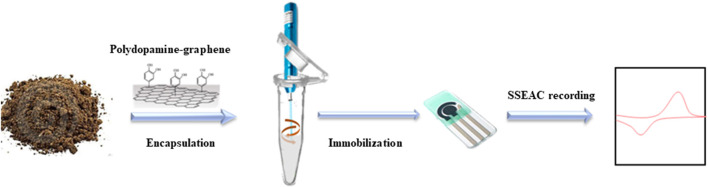
Schematic process of proposed electrochemical method for soil particle encapsulation.

The underlying technology of SSEAC can be traced back to a series of original works by Scholz's team in the late 1980s and early 1990s (Scholz et al., 1989a, 1989b; Scholz and Lange, 1992). The first analysis of solid particles based on the SSEAC technique was also presented by Scholz's team in 1995 (Scholz et al., 1995). They found a positive correlation between the current intensity of the electrochemical oxygen precipitation reaction and the amount of radiation accumulated on ceramic particles. In the last decade, the Domenech-Carbo team combined metal corrosion and electrochemical redox mechanisms and developed several methods for the analysis of metal particles including electrochemical impedance spectroscopy (Doménech-Carbó et al., 2014), Tafel curves (Doménech-Carbó et al., 2016) and polarization curves (Doménech-Carbó et al., 2012). They also proposed voltammetry of immobilized microparticles (VIMP) for the rapid and effective electrochemical analysis of solid particles. SSEAC analysis is a promising technique for soil analysis due to its low cost and speed, minimal sample requirement, and applicability to both inorganic and organic particles. However, due to technical constraints, a large number of SSEAC assays based on the VIMP method have a large uncontrollability at both the electrical and sample of the testing process. Therefore, we thought to encapsulate soil particles with polydopamine-modified graphene, which has high specific surface, high electrical conductivity and good immobilization ability. The material was used to adsorb oxidizable organic compounds from the soil particles with high efficiency. The encapsulated soil particles were directly immobilized on the surface of a printed electrode with an integrated three-electrode system for highly sensitive signal acquisition, as shown in Figure 1.

This work proposes to perform rapid detection of organic matter content of soil by detecting the reaction of oxidizable organic compounds in soil samples with reactive oxygen species (ROS) generated by *in situ* electrochemistry as an indication. Soil particles were adsorbed and coated with polydopamine-modified graphene and cemented on the electrode surface. This allows for signal acquisition of oxidizable organic compounds in the soil sample. We also investigated the electrochemical response mechanism of soil particles under encapsulation. A reasonable signal acquisition standard was investigated, and the correspondence between the oxidizable organic compounds in soil particles and the overall organic matter content was explored by using the conventional potassium dichromate external heating method and NIR detection method as controls.

**FIGURE 2:**
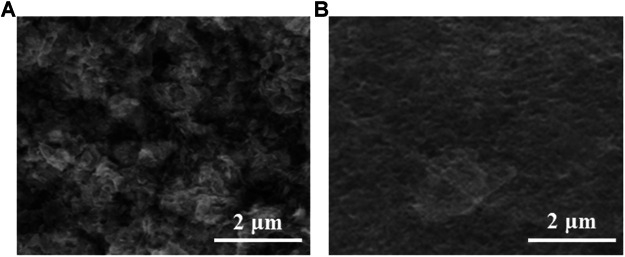
SEM images of **(A)** soil particles and **(B)** polydopamine-graphene encapsulated soil particles on the electrode surface.

Modification of graphene surfaces using polydopamine is often used to improve the dispersion of graphene in the aqueous phase and to achieve efficient modification of other material surfaces with the help of the adhesion of polydopamine. The SEM images in Figure 2 show the soil particles directly immobilized on the electrode surface and the soil particles after polydopamine-graphene coating. It can be seen that the polydopamine-graphene can coat the soil particles very well from the microscopic point of view, and has good film formation on the electrode surface to achieve stable immobilization. This makes the soil particles not easily detach from the electrode during the electrochemical detection process and improves the accuracy of the detection.

**FIGURE 3:**
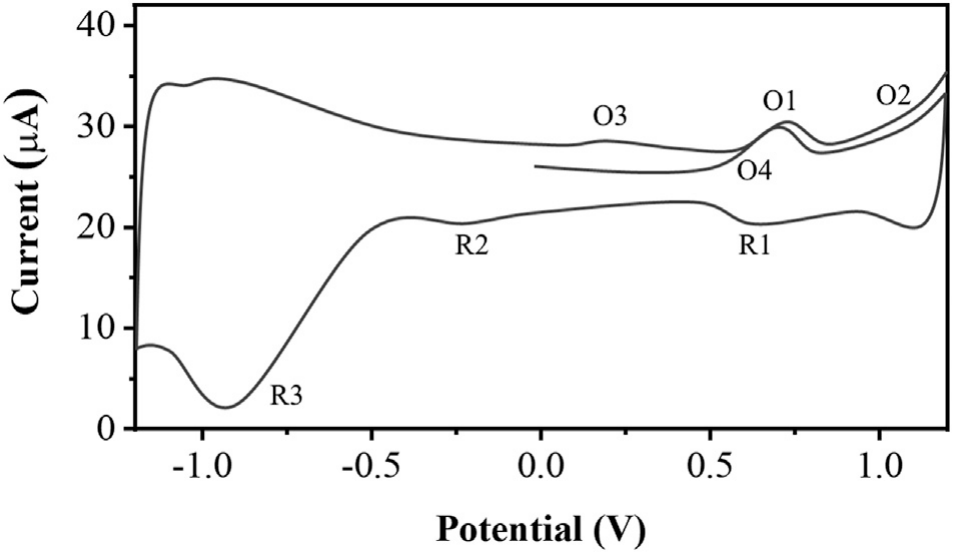
SSEAC spectra of soil particles.

The cyclic voltammogram of the soil particles after half-derivative convolution is shown in Figure 3. There are multiple oxidation peaks (O1, O3, O4) in the range of +0.2 to +0.8 V at the anode. From +1.0 V onwards there is a significant current rise due to oxygen precipitation. During the negative sweep, there is a clear reduction peak at +0.60 V (R1), followed by a series of weak overlapping reduction peaks between +0.2 and 0.6 V (R2). A distinct reduction peak occurs near about −1.0 V due to hydrogen precipitation. These oxidation and reduction peaks are signals due to the electrochemical oxidation or reduction of organic matter in the soil. These observed processes may be due to the superposition of signals from a considerable number of compounds. Nevertheless, if the electrochemical redox fingerprints of lignin and different organic compounds are compared, it is shown that the O1 process is probably the oxidation of the catechol moiety to the corresponding o-quinones. O2 is the oxidation of monophenolic moiety and/or methoxyphenol (Milczarek, 2009; Admassie et al., 2014). R1 is the reduction process of some of the substances in O1 in the cathodic scan, while the R2 process is mainly attributed to the reduction of organic compounds with a quinone structure. During the second anodic scan, the peaks of O1 and O2 were reduced. A series of additional oxidation signals (O3) between −0.1 and +0.2 V appeared, suggesting the generation of new reactive oxygen clusters during the electrochemical process.

We tried to divide the collected soil samples into sample sets, of which 30 samples were the test set and 47 samples were the modeling set. In the modeling set, we divided 23 of the samples into calibration set and 24 samples into prediction set in order to avoid distortion of model evaluation as well as to get stable prediction. In order to better analyze the models built by the EC-PLS method, we selected the first anode scan 0.0–1.0 V and cathode scan 0.7 to −1.0 V, the second anode scan 0.0–1.0 V and the full voltammetric region to build the PLS models, respectively. Table 1 shows a summary of the optimal PLS model results for different voltammetric regions, from which it can be seen that the best modeling results were obtained for the full voltammetric region, followed by very close modeling results under the anodic scan, indicating that the SSEAC information of oxidizable compounds in the soil can correspond better to the amount of organic matter content in the soil.

**TABLE 1:** Parameters and prediction results of the optimal PLS voltammetric region model for soil SSEAC data.

Location	Region	*F*	SEP^+^	SEP_Ave_	SEP_SD_	R_P,Ave_	R_P,SD_
A	0.0–1.0 V	9	0.277	0.271	0.022	0.917	0.016
	0.7–1.0 V	6	0.322	0.299	0.021	0.903	0.009
	0.0–1.0 V	9	0.260	0.241	0.014	0.922	0.007
	Whole scan	11	0.247	0.230	0.022	0.919	0.011
B	0.0–1.0 V	13	0.857	0.779	0.057	0.869	0.021
	0.7–1.0 V	17	1.207	1.035	0.124	0.801	0.043
	0.0–1.0 V	17	0.754	0.576	0.155	0.933	0.033
	Whole scan	26	0.611	0.471	0.151	0.951	0.031
C	0.0–1.0 V	20	0.365	0.318	0.041	0.814	0.022
	0.7–1.0 V	29	0.418	0.366	0.061	0.851	0.041
	0.0–1.0 V	17	0.221	0.201	0.020	0.923	0.007
	Whole scan	26	0.209	0.191	0.022	0.955	0.009

From Table 1, it can be seen that the three locations of soil samples are best modeled in the whole scan range. However, the number of potentials in the whole scan range in the full CV scan is very high and therefore the model is very complex. The modeling effect of 0–1 V is very close to the whole scan range, which indicates that the information potential of the soil is mainly in this potential window. Therefore, we modeled 0–1 V has been selected for EC-PLS model establishment for three locations of soil samples. Table 2 shows the parameters and prediction effects of the optimal EC-PLS models for three locations of soils.

**TABLE 2:** The parameters and prediction effects of the optimal EC-PLS models for three locations of soils.

Location	Potential (V)	*G*	*N*	*F*	SEP^+^	SEP_Ave_	SEP_50_	R_p_,_Ave_	R_P,SD_
A	0.204	47	10	4	0.248	0.233	0.015	0.922	0.006
B	0.306	33	3	11	0.551	0.502	0.044	0.941	0.006
C	0.267	31	9	17	0.206	0.191	0.013	0.950	0.005

From Table 2, the optimal EC-PLS model for the soil samples at location A has a starting potential *I*, number of potentials *N*, and number of potential intervals *G* of 0.204 V, 47, and 10, respectively, with corresponding SEP^+^ and R_P,Ave_ were 0.248 wt% and 0.922, respectively. The optimal EC-PLS models for the soil samples from location B corresponded to a starting potential *I*, number of potentials *N*, and number of potential intervals *G* of 0.306 V, 33, and 3, respectively. The corresponding SEP^+^ and R_P,Ave_ were 0.551 wt% and 0.941, respectively. The optimal EC-PLS model for the soil samples at location C had starting potentials *I*, number of potentials *N* and number of potential intervals *G* of 0.267 V, 31 and 9, respectively. Because different soils contain different components with different information potential intervals (Rasmussen et al., 2018; Liang et al., 2019), the electrochemical voltammogram analysis model differs for different types of soil organic matter.

**FIGURE 4:**
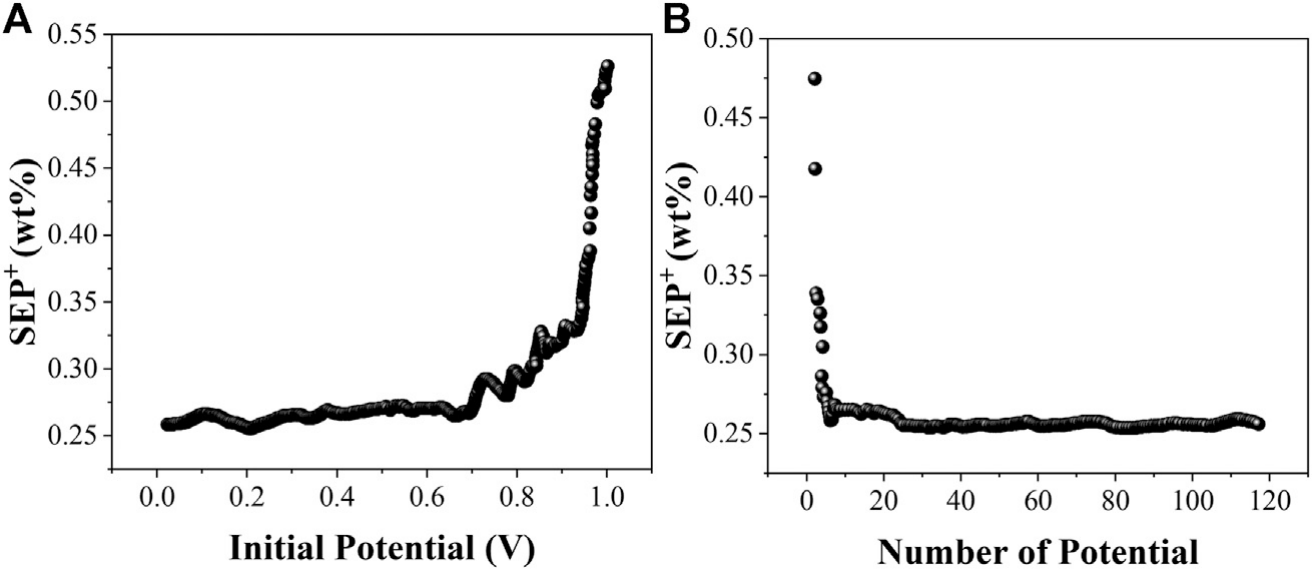
The optimal SEP^+^ corresponding to the starting potential *I* and the number of potentials *N* of the soil EC-PLS model for A location.

**FIGURE 5:**
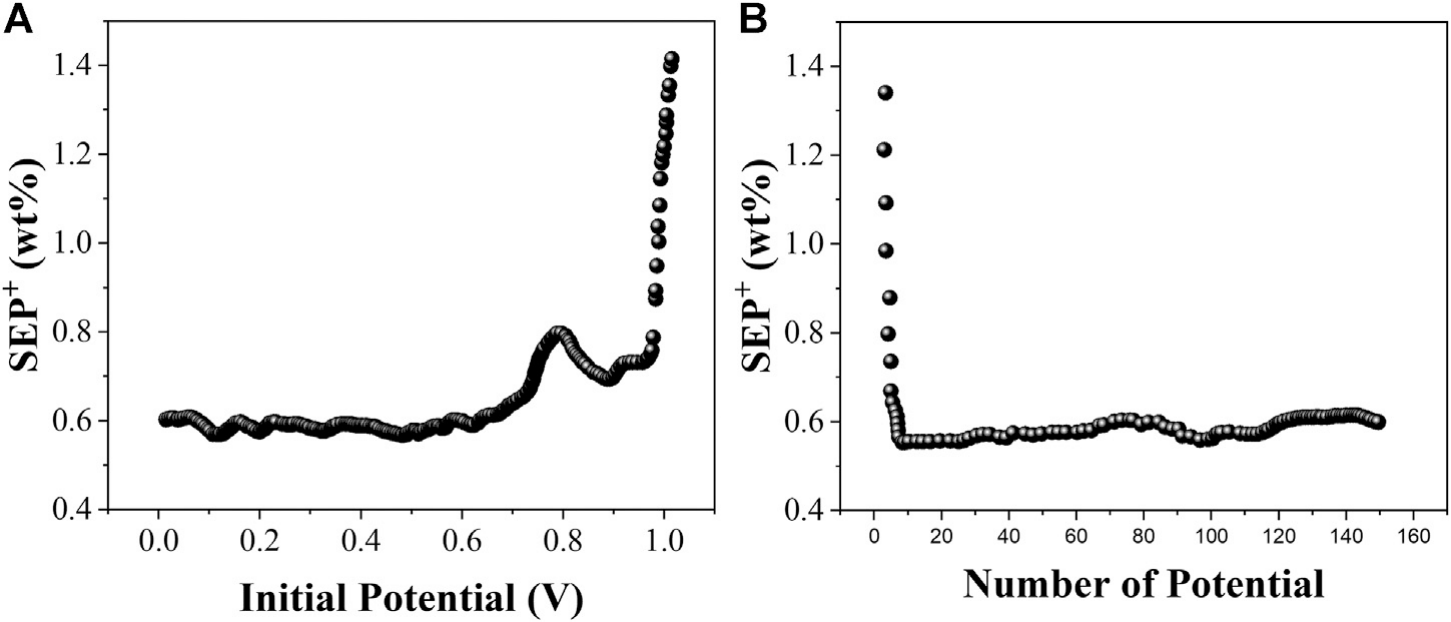
The optimal SEP^+^ corresponding to the starting potential *I* and the number of potentials *N* of the soil EC-PLS model for B location.

**FIGURE 6:**
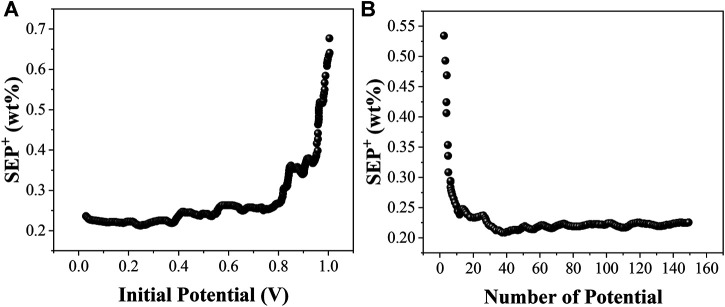
The optimal SEP^+^ corresponding to the starting potential *I* and the number of potentials *N* of the soil EC-PLS model for C location.

In order to examine the effect of different information potentials on the model prediction, we fix one of the parameters while varying the remaining parameters and filter the locally optimal models based on the minimum SEP^+^ values. In this case, by fixing the potential *I* and varying the remaining parameters, the locally optimal model corresponding to each starting potential *I* is filtered according to the minimum SEP^+^ value. Similarly, by fixing the number of potentials *N* and varying the remaining parameters, the local optimum model corresponding to each number of potentials *N* and the corresponding combination of the remaining parameters can be filtered. The EC-PLS local optimum models corresponding to the starting potentials and the number of potentials for the electrochemical analysis models of organic matter from soils at sites A, B and C are shown in Figure 4, Figure 5 and Figure 6, respectively.

The EC-PLS local optimum model for the organic matter of site A has a starting potential *I* and a number of potentials *N* of 0.204 V and 47, respectively, and the EC-PLS local optimum model for the organic matter of site B has a potential wavelength *I* and a number of potentials *N* of 0.306 V and 33, respectively, as seen in Figure 4. Based on the previous conclusions, we know that the EC-PLS model for soils with a smaller number of potentials has a much lower model complexity, which helps to improve the model prediction. Moreover, the EC-PLS wavelength models of soils at all three sites are better than the PLS wavelength models of the whole scan range.

## Conclusion

The detection of organic matter in the soil allows us to accurately grasp the dynamics of soil fertility, which is important for improving the utilization of agricultural resources and the modern management of agriculture. In this work, we propose an alternative approach to evaluate the organic matter content of undigested/dissolved soil samples directly by using the SSEAC technique for rapid detection. Soil particles contain a variety of plant-derived organic matter molecules, some of which are capable of electrochemical oxidation and/or reduction reactions, such as indoles, polyphenols and quinones. The encapsulated soil particles were directly immobilized on the surface of a printed electrode with an integrated three-electrode system for highly sensitive signal acquisition. Analytical models were developed for the analysis of soil organic matter content at each of the three sites based on different modeling approaches.

## Data Availability

The original contributions presented in the study are included in the article/supplementary material, further inquiries can be directed to the corresponding author.
